# Supplementary material for: In-organoid single-cell CRISPR screening reveals determinants of hepatocyte differentiation and maturation
Source: Genome Biol. 2023 Oct 31;24:251. doi: 10.1186/s13059-023-03084-8 (PMC10617096; doi:10.1186/s13059-023-03084-8)
Supplement: Supplementary file 1 — Additional file 1: Fig. S1. Comparison of different differentiation strategies for mICOs. mICOs isolated from livers of spCas9-EGFP knock-in mice were cultured under DM for differentiation or EM for expansion for 7 or 12 days. Cultures were harvested for transcriptional profiling. a qRT-PCR analysis showing relative gene expression as mean ± s.e.m. (n = 4) of known hepatocyte markers (Alb, Ttr, Cyp3a11, Apoa1, Mup20, Mrp2, Sutl1a1, and Aldh1a1) or biliary duct markers (Sox9 and Spp1) for mICO cultures maintained under expansion medium (EM) or transferred to differentiation medium (DM) for 7 or 12 days (DM_7 or DM_12). Following one-way ANOVA, pairwise comparisons were performed using the Tukey-HSD test. Only statistically significant comparisons (P-value <0.05) were marked. b Principal-component analysis (PCA) of transcriptional profiles of the samples under each condition. X-axis: the principal component with the largest explanatory variance. Y-axis: the principal component with the second largest explanatory variance. c Venn diagram showing the overlap of upregulated and downregulated genes in DM_7 and DM_12 groups compared to the EM group. d The top 5 gene ontology biological processes enriched for upregulated or downregulated genes shared in DM_7 and DM_12 groups. e GSEA of transcriptional profile using KEGG gene sets of MsigDB. Bubble size indicates -log10 (FDR q-value) and the color of bubble denotes normalized enrichment score (NES). f-h Enrichment score (ES) plots displaying the top 3 pathways of the most highly enriched gene sets including retinol metabolism (f), drug metabolism cytochrome p450 (g), and complement and coagulation cascades (h). The statistically significance threshold was set to 0.05 and the adjusted method used was the q-value. Fig. S2. sgRNA assignments and scRNA-seq quality control in pilot CROP-seq screen. a The distribution of the number of sgRNAs detected per cell in EM and DM group, respectively. b Violin plots of the number of genes [file 13059_2023_3084_MOESM1_ESM.docx]

**Supplemental figures**


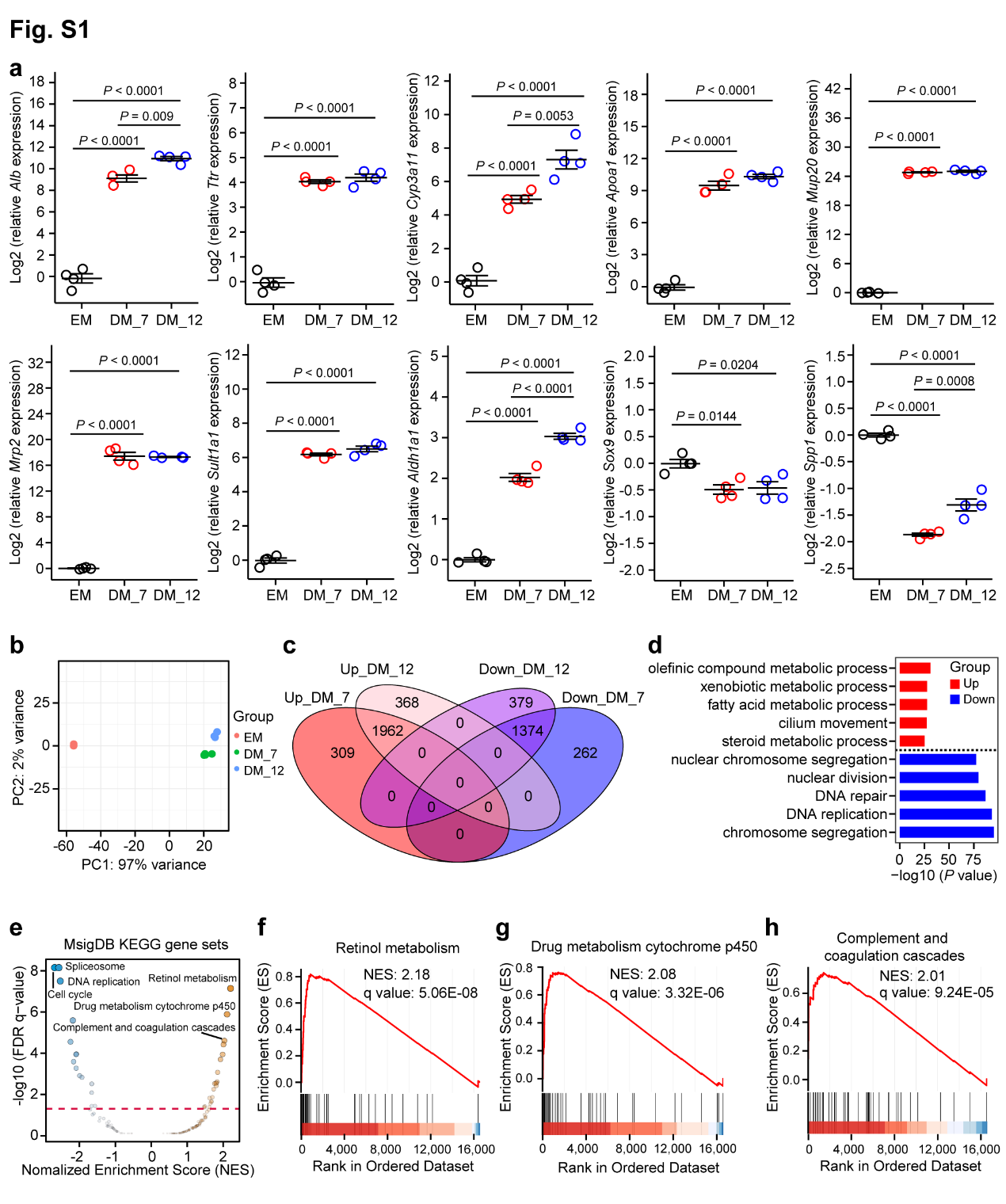


**Fig. S1 Comparison of different differentiation strategies for mICOs.** mICOs isolated from livers of spCas9-EGFP knock-in mice were cultured under DM for differentiation or EM for expansion for 7 or 12 days. Cultures were harvested for transcriptional profiling. **a** qRT-PCR analysis showing relative gene expression as mean ± s.e.m. (n = 4) of known hepatocyte markers (*Alb*, *Ttr*, *Cyp3a11*, *Apoa1*, *Mup20*, *Mrp2*, *Sutl1a1*, and *Aldh1a1*) or biliary duct markers (*Sox9* and *Spp1*) for mICO cultures maintained under expansion medium (EM) or transferred to differentiation medium (DM) for 7 or 12 days (DM_7 or DM_12). Following one-way ANOVA, pairwise comparisons were performed using the Tukey-HSD test. Only statistically significant comparisons (*P*-value <0.05) were marked. **b** Principal-component analysis (PCA) of transcriptional profiles of the samples under each condition. X-axis: the principal component with the largest explanatory variance. Y-axis: the principal component with the second largest explanatory variance. **c** Venn diagram showing the overlap of upregulated and downregulated genes in DM_7 and DM_12 groups compared to the EM group. **d** The top 5 gene ontology biological processes enriched for upregulated or downregulated genes shared in DM_7 and DM_12 groups. **e** GSEA of transcriptional profile using KEGG gene sets of MsigDB. Bubble size indicates -log10 (FDR *q*-value) and the color of bubble denotes normalized enrichment score (NES). **f-h** Enrichment score (ES) plots displaying the top 3 pathways of the most highly enriched gene sets including retinol metabolism (**f**), drug metabolism cytochrome p450 (**g**), and complement and coagulation cascades (**h**). The statistically significance threshold was set to 0.05 and the adjusted method used was the *q*-value.


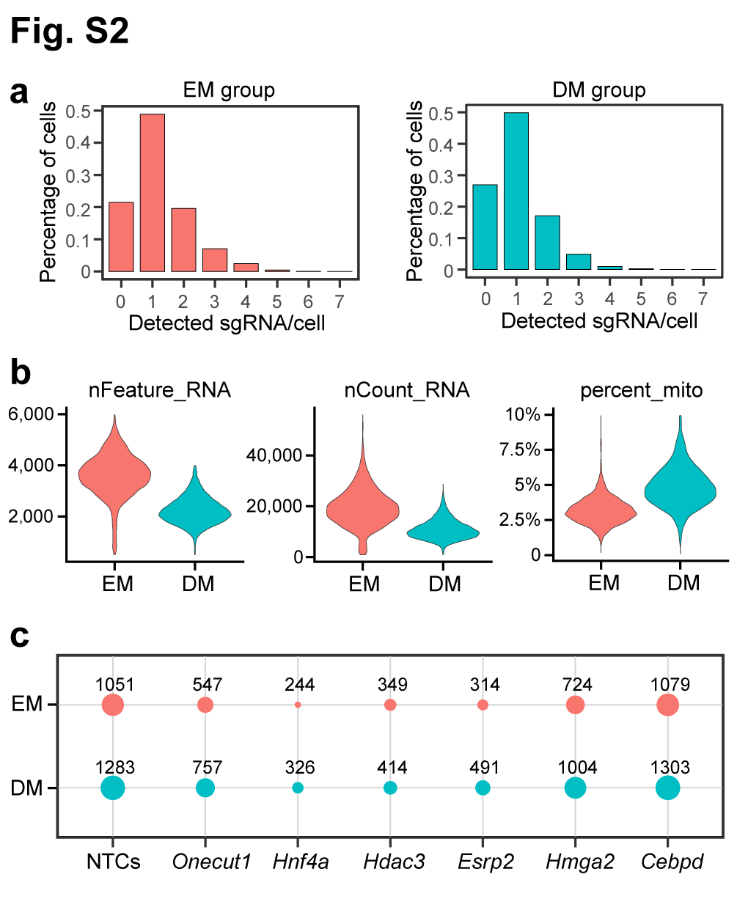


**Fig. S2 sgRNA assignments and scRNA-seq quality control in pilot CROP-seq screen. a** The distribution of the number of sgRNAs detected per cell in EM and DM group, respectively. **b** Violin plots of the number of genes (feature), number of UMIs (count) and mitochondrial gene percentages for the cells with unique sgRNA assignments in EM or DM group. Cells with more than 200 expressed genes and less than 6,000 expressed genes as well as less than 10% mitochondrial genes were retained. **c** The number of cells expressing a unique sgRNA targeting the same gene.


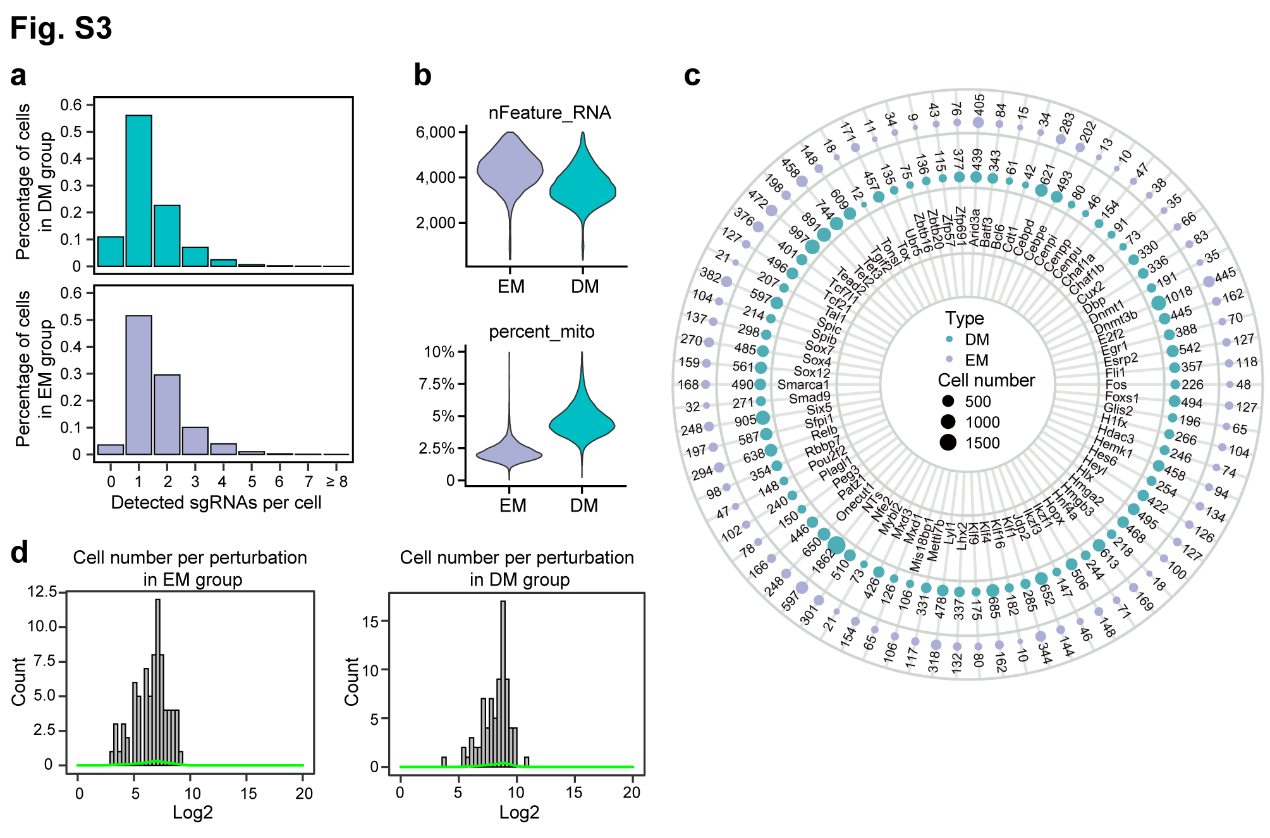


**Fig. S3 sgRNA assignments and scRNA-seq quality control in CROP-seq screen. a** Distribution of the number of sgRNAs detected per cell in EM and DM groups, respectively. **b** Quality controls of CROP-seq screen, with the same criteria as that in the pilot study. **c** Number of single cells expressing a unique sgRNA for each targeting gene. **d** Distribution of cell numbers for each perturbation in EM (left) and DM (right) groups.


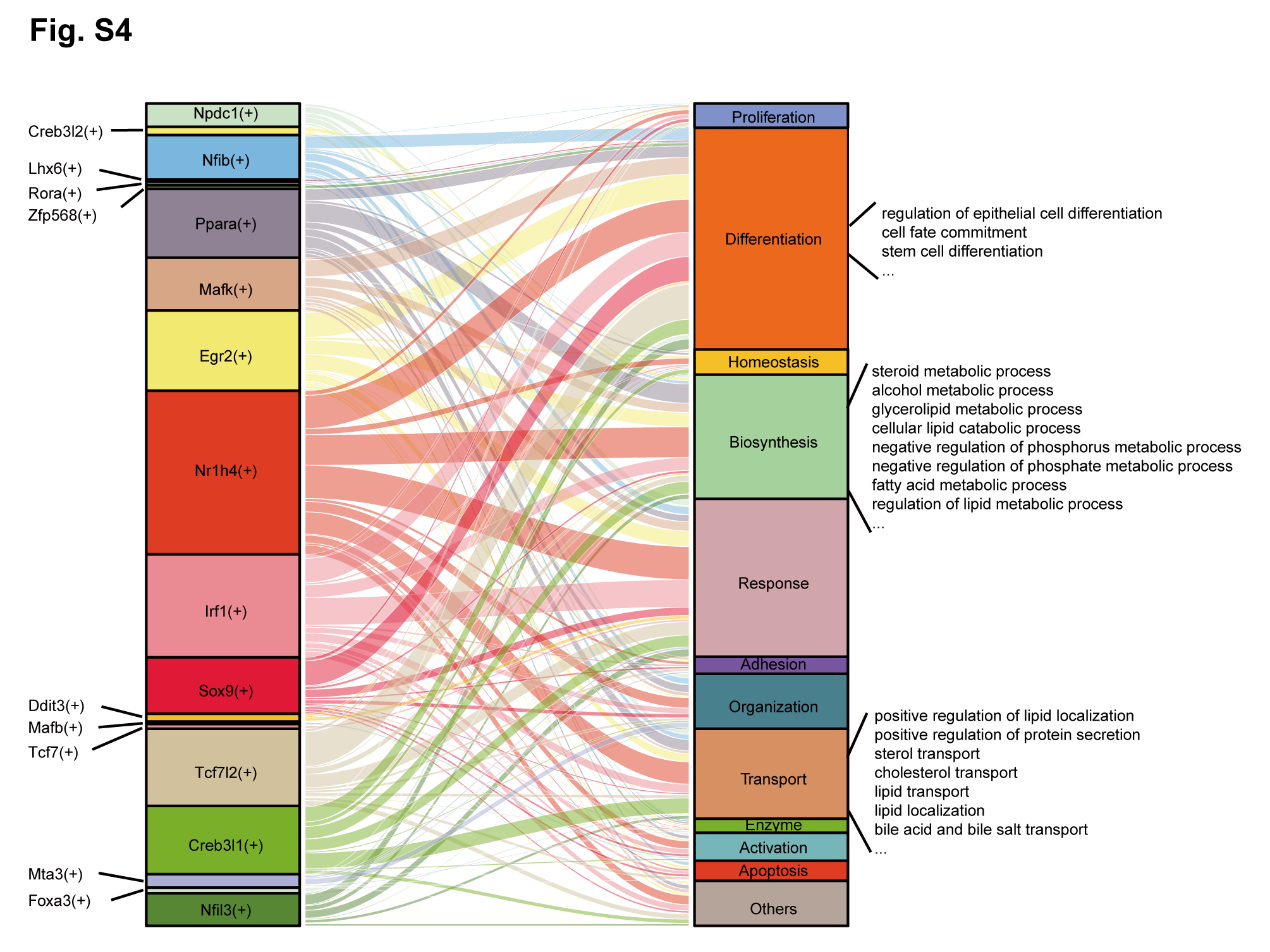


**Fig. S4 Sankey plot of enrichment analysis for the top 20 variable regulons.**

The first column: Top 20 variable regulons (number of terms > 0). The second column: clusters of GO-BP terms (adjusted *P*-value using BH methods < 0.05). The third column: the focused terms. The edge linking the first column to the second column: the term *i* of regulon *j* is the member of cluster *k*. The thickness of edge: the number of terms.


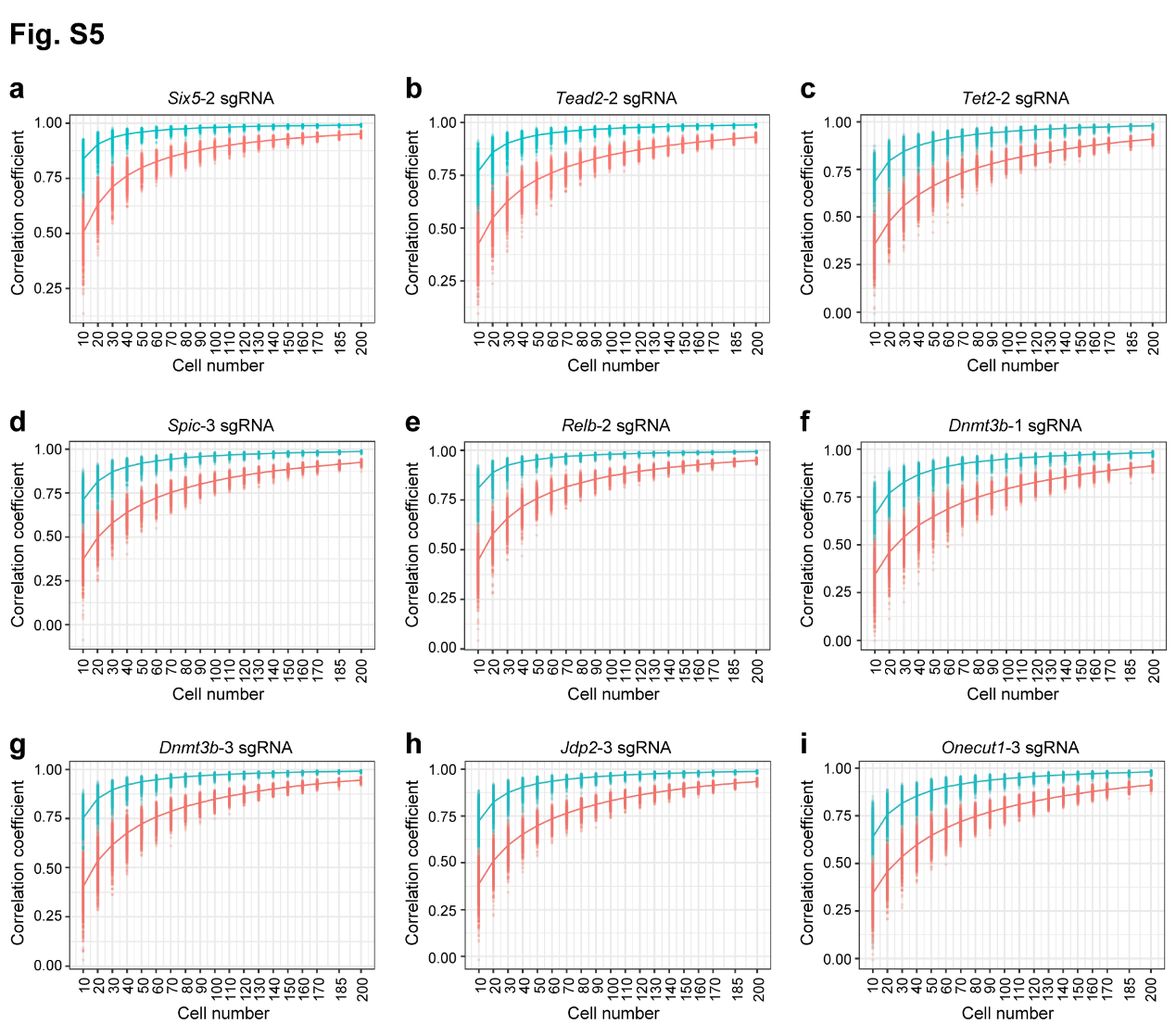


**Fig. S5 Evaluating the number of cells required for robustly measuring the regulon activities and expression.** **a-i** Scatter plot showing the Spearman’s correlations between average regulon activities (blue points) or average gene expression (red points) in the indicated number of cells and that in the whole sample. The cells were randomly sampled 1000 times for each indicated cell number. The curves represent the median point of the 1000-time iterations. Cells with sgRNAs targeting *Six5* (**a**), *Tead2* (**b**), *Tet2* (**c**), *Spic* (**d**), *Relb* (**e**), *Dnmt3b* (**f** and **g**), *Jdp2* (**h**), *Onecut1* (**i**) were shown.


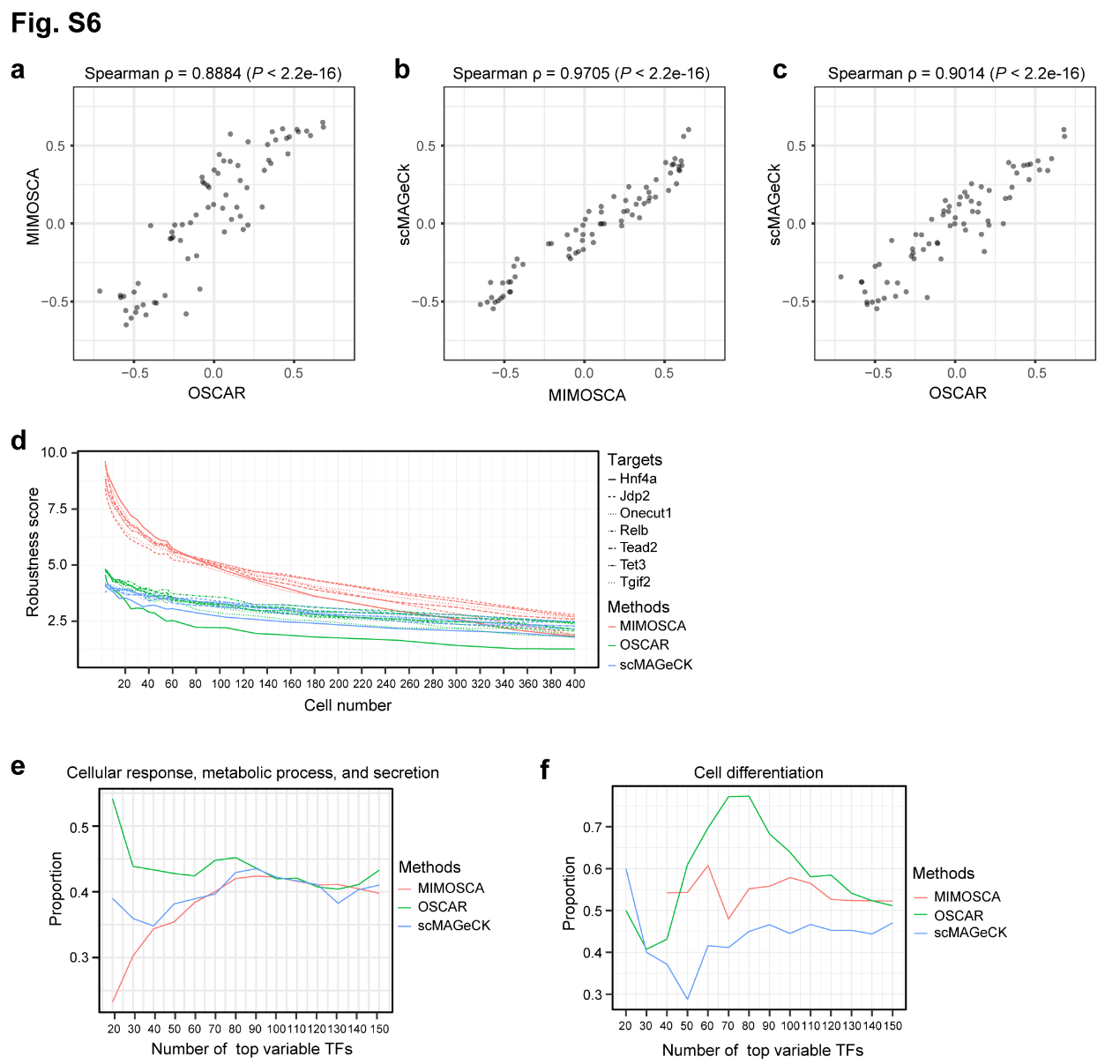


**Fig. S6 Comparison of the three methods on the performance of mapping perturbation effects.** **a-c** Scatter plots showing the correlation of perturbation effects among the three methods. Each point represents the perturbation effect of a target relative to *Hnf4a*, which is calculated as the Spearman’s correlation coefficient between the top 200 variable features for that target and those for *Hnf4a*. **d** The line plot shows the robustness of perturbation effects identified by the three methods by subsampling different numbers of cells. The robustness score represents the agreement of coefficients calculated from the 100 subsets of data with those calculated from the whole dataset. **e, f** The line plot shows the proportion of the key GO term module associated with different numbers of top variable TFs identified by the three methods, using our OSCAR dataset (**e**) or the ESC CROP-seq data (**f**).


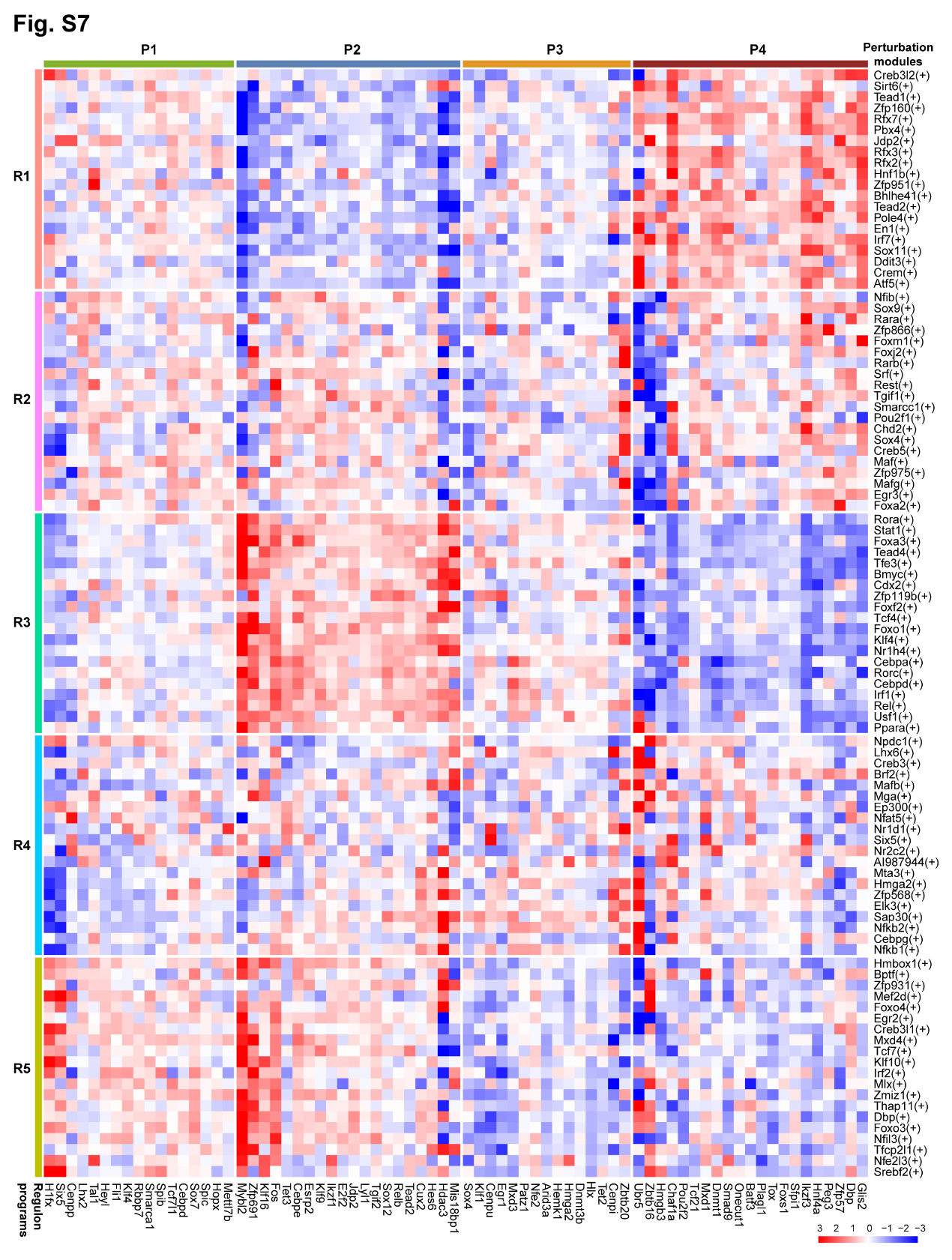


**Fig. S7 Association of each perturbed gene with the activities of top 20 variable regulons in each regulon program.** Column: each perturbed gene from P1-P4 perturbation groups. Row: top 20 variable regulons for each regulon program.


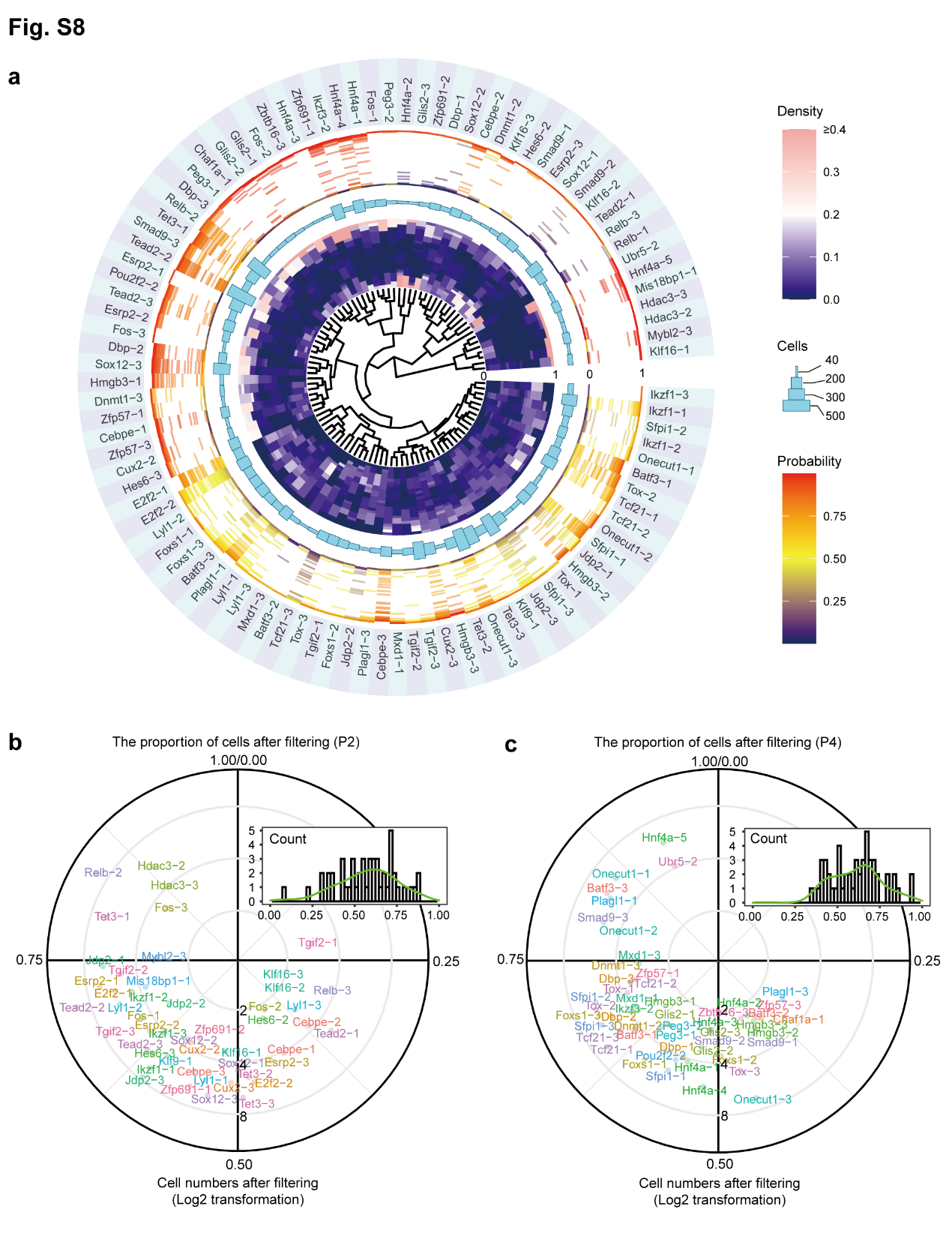
**Fig. S8 Filtering unperturbed cells from scRNA-seq data by MIMOSCA and Gaussian fitting. a** Distribution of perturbation probability of each sgRNA before filtering. The innermost layer: hierarchical clustering tree indicating the similarity of the distribution of perturbation probability calculated by MIMOSCA. The second layer: annular heatmap showing the relative density (, where *Count_i_* denotes the number of cells within bin*_i_*, and the width of each bin is 0.05) of each sgRNA. The third layer: annular bar plot showing the number of cells before filtering. As a reference, the number of cells with sgRNA Sfpi1−1 is 183. The fourth layer: annular band plot showing the performance of GMM. From inside to outside of the fourth layer, the location of bands indicates the probability of each cell being assigned to the second cluster (Higher probability of perturbation) of the GMM in each bin, and the color of bands indicates the probability of perturbation of each cell. **b**, **c** The angle of the polar coordinate plot represents the proportion of cells retained, and the distance from the pole represents the number of cells retained (log2 transformation). Histogram showing the distribution of the proportion of cells retained for Module P2 (**b**) or Module P4 (**c**).


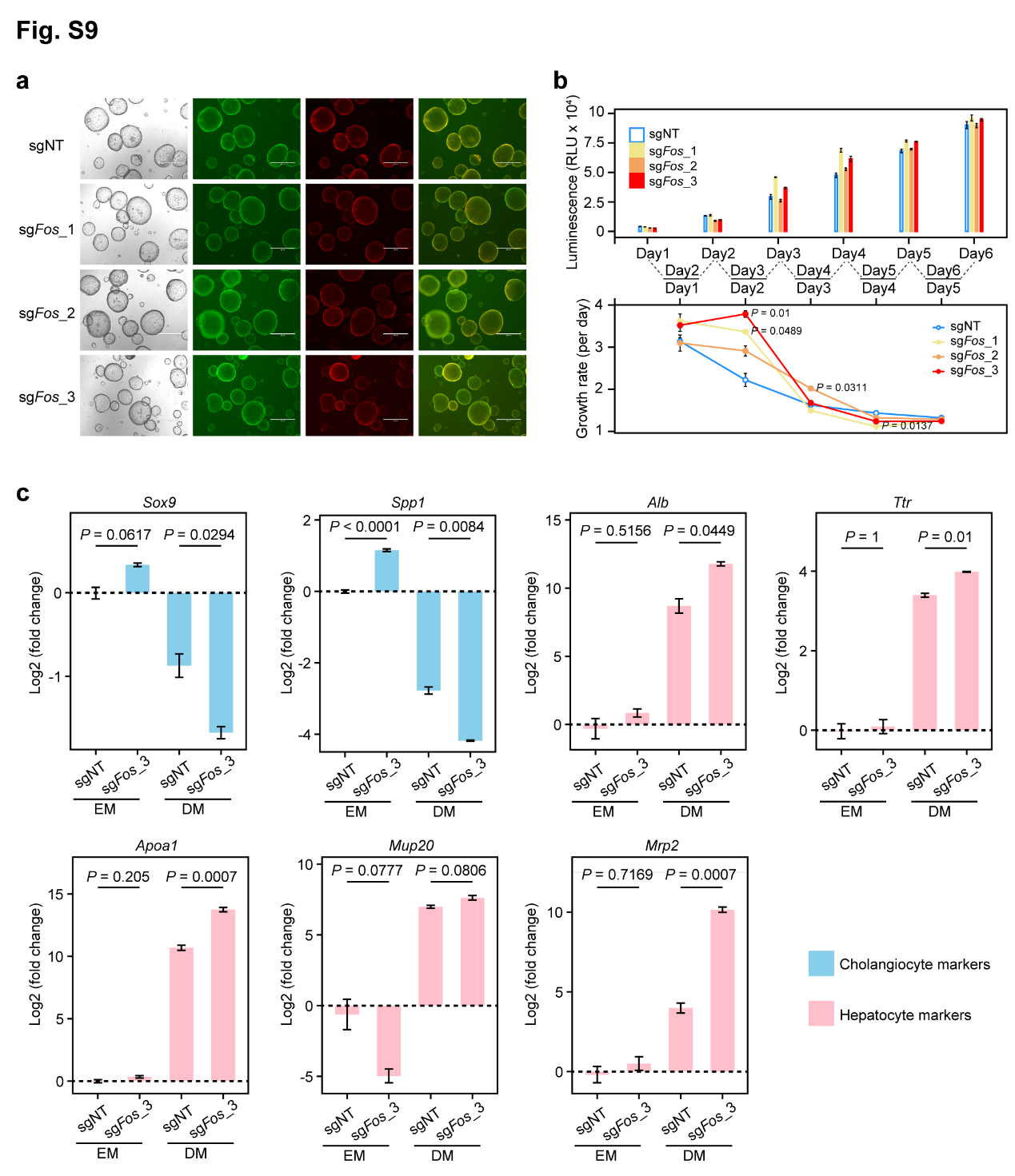


**Fig. S9 Comparison of growth and gene expression in Fos-KO and control mICOs under expansion or differentiation medium.**

**a** Representative images showing cyst structures of mICOs stably expressing either non-targeting sgRNA (sgNT) or sgRNA targeting *Fos* (sg*Fos*_1, sg*Fos*_2, sg*Fos*_3). Scale bars: 500 μm. **b** Upper panel: Growth of three *Fos*-KO lines and control mICOs in EM condition was monitored using the Cell-titer Glo assay. Luminescence values were taken every 24 hours. Error bars indicate the standard error of the mean. Lower panel: Growth rates for the *Fos*-KO lines and control mICOs in expansion medium (EM) were determined over 24-hour intervals across a 6-day period. Growth rates were calculated by dividing each day's luminescence value by the previous day's average. Error bars represent standard errors of the mean (n = 3). Statistical significance was tested using one-way ANOVA, followed by Bonferroni’s correction for pre-planned pairwise comparisons between sgNT and each sg*Fos* line. A *P*-value less than 0.05 was considered statistically significant, and only such comparisons are marked on the graph. **c** qRT-PCR analysis of the relative gene expression of selected cholangiocyte markers (*Sox9* and *Spp1*) and hepatic markers (*Alb*, *Ttr*, *Apoa1*, *Mup20*, and *Mrp2*) in mICO cultures. Organoid cultures were maintained under expansion medium (EM) or switched to differentiation medium (DM) for 7 days. Statistical significance was assessed using one-way ANOVA, followed by Bonferroni’s correction for pre-planned pairwise comparisons between sgNT and sg*Fos*_3 under both EM and DM conditions. A *P*-value less than 0.05 was considered statistically significant.


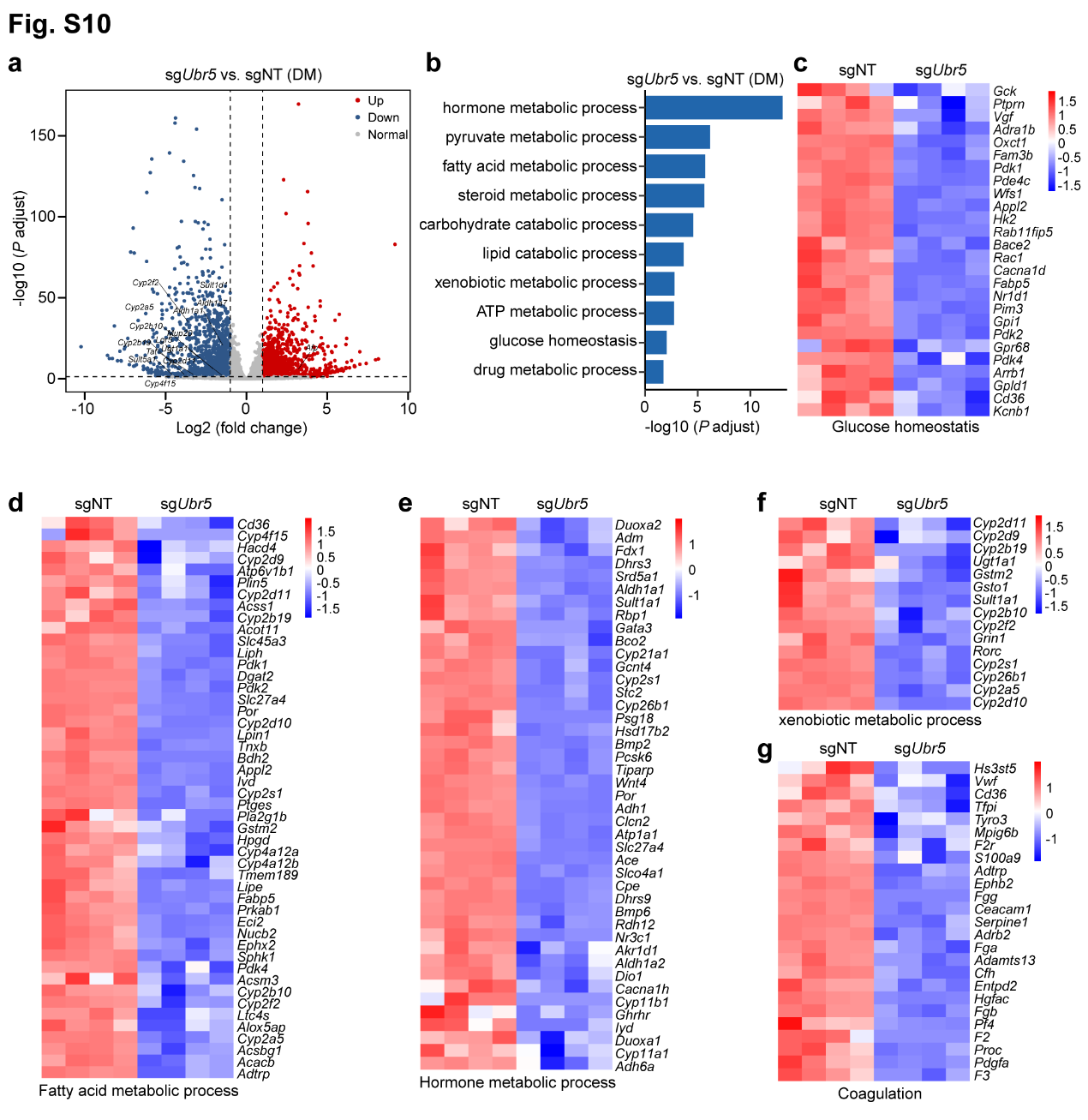


**Fig. S10 *Ubr5* depletion in mICOs weakens hepatocyte differentiation and maturation.** *Ubr5* KO mICOs and the NT controls were maintained under DM condition for differentiation with the strategy as illustrated in Fig. 3a. **a** Volcano plot shows differentially expressed genes (|log2FoldChange| < 1 and an adjusted *P*-value using the BH methods < 0.05). Blue, downregulated genes; red, upregulated genes. Representative markers were labelled. **b** Selected GO terms significantly enriched for genes down-regulated in *Ubr5* KO cultures. **c-g** Heatmaps showing differentially expressed genes involved in glucose homeostasis (**c**), fatty acid metabolic process (**d**), hormone metabolic process (**e**), xenobiotic metabolic process (**f**) and coagulation (**g**).

**
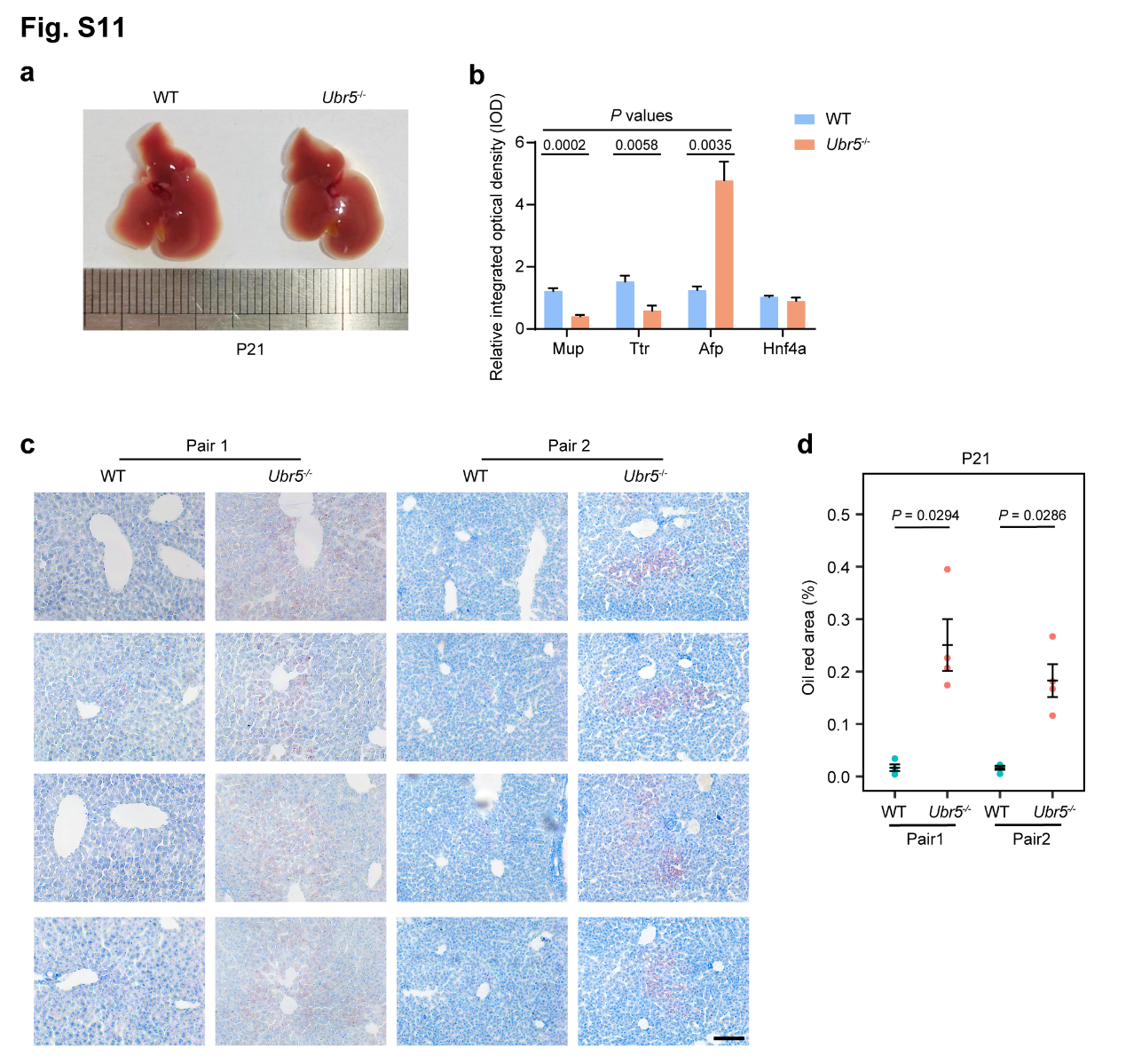
**

**Fig. S11 *Ubr5* depletion *in vivo* weakens hepatocyte differentiation and maturation. a** Morphology of WT and *Ubr5*-KO livers from mice at P21. Scale bars: 100 μm. **b** Quantification of IHC images by calculating the relative Integrated Optical Density (IOD). Data are represented as mean ± s.e.m. (n = 5) and were compared by the two-tailed student’s *t* test. **c** Representative images of Oil Red O staining of WT and *Ubr5*-KO livers from two pairs of mice at P21. Scale bars, 100 μm. **d** Percentages of lipid droplets area in (**c**) were shown as mean ± s.e.m. (n = 4) and compared by Wilcoxon rank sum test within each pair. A *P*-value less than 0.05 was considered statistically significant.

**Fig. S12** **Uncropped images of Western blots. a** Western blot gel images for FOS and β-actin; boxes indicate the cropped regions in Fig. 4b. **b** Images of western blot gel for UBR5 and Vinculin; the cropped regions in Fig. 5c are indicated by the boxes.
